# Amyand’s hernia: a case of right inguinoscrotal hernia with appendiceal content

**DOI:** 10.1097/MS9.0000000000002137

**Published:** 2024-05-06

**Authors:** Shreya Khandelwal, Alamjeet Kaur, Shashi Singh, Aneek Ghosh

**Affiliations:** a Koirala Institute of Health Sciences, Dharan; b Kharkiv National Medical University; c Tribhuvan University, Kathmandu; d Kolkata Medical College, Nepal

**Keywords:** Amyand’s hernia, appendicitis, diagnostic challenges, inguinoscrotal hernia, surgical intervention

## Abstract

**Introduction:**

Amyand’s hernia, a rare condition where the appendix becomes lodged in the inguinal canal, poses diagnostic challenges due to its varied clinical presentations and lack of distinctive radiological features. This case underscores the importance of early detection and comprehensive diagnostic evaluation.

**Case Presentation:**

A 30-year-old male presented with a 2-year history of right inguinoscrotal swelling, culminating in agonizing symptoms and irreducible masses over the inguinoscrotal area. Despite the absence of significant medical history, diagnostic imaging confirmed a complete right inguinoscrotal hernia and bilateral hydrocele with internal echoes.

**Clinical Discussion:**

The case illustrates the difficulties in preoperative diagnosis of Amyand’s hernia, emphasizing the reliance on imaging modalities and clinical assessment. Successful surgical intervention involving appendectomy and hernioplasty highlights the necessity for prompt diagnosis and management.

**Conclusion:**

This case exemplifies the challenges and complexities associated with Amyand’s hernia, emphasizing the importance of early recognition and comprehensive surgical planning. Moving forward, increased clinical vigilance and awareness are essential to ensure optimal patient outcomes in cases of inguinoscrotal pathology.

## Introduction

Highlights

- Amyand’s hernia comprises <1% of inguinal hernias, necessitating increased research.
- Preoperative identification is complex, often resembling incarcerated hernias.
- Appendectomy and hernioplasty demonstrate effective surgical management.
- Computed tomography and ultrasound assist in diagnosis, especially in abdominal discomfort with a groin mass.
- Surgical decisions consider preventive appendectomy and evolving techniques.
- Further studies essential to reduce misdiagnosis and improve outcomes.

A hernia is characterized by the protrusion of an organ or its fascia through the wall of a cavity, with the risk of complications such as incarceration and strangulation^1^. Ventral abdominal and inguinal hernias usually involve the bowel or omentum, but it is rare for the appendix to be within the herniated area^2^. The occurrence of this rare phenomenon ranges from 0.19 to 1.7% of all hernias and is referred to as Amyand’s hernia^3,4^, honoring Claudius Amyand, who performed the first successful appendectomy during the treatment of an 11-year-old boy who presented with a right inguinal hernia^5^.

Amyand’s hernia presents diagnostic challenges due to its vague symptoms and the absence of clear radiographic findings, often resulting in misdiagnosis as a strangulated hernia. Diagnosis typically occurs incidentally during surgery, which complicates operative management. The estimated incidence of appendicitis within an inguinal hernia range from 0.07 to 0.13%, while the estimated incidence of a perforated appendix within an inguinal hernia represents 0.1% of all appendicitis cases demonstrating the potential severity of this condition^6–8^. Mortality rates associated with Amyand’s hernia vary significantly, ranging from 14 to 30%, with a notable correlation observed between the condition and the dissemination of sepsis throughout the peritoneum^9^. Despite its rarity, Amyand’s hernia emphasizes the critical importance of meticulous diagnostic assessment and prompt surgical intervention to mitigate potentially life-threatening consequences, including perforation, strangulation, or the development of appendicitis^8^.

This article presents a case involving a 30-year-old man who exhibited a right-sided inguinal hernia, later identified intraoperatively as Amyand’s hernia. A concise review of the clinical presentation, diagnosis, and treatment approach for this specific instance of Amyand’s hernia is provided. In accordance with the SCARE 2023 criteria, this case report meticulously offers a comprehensive overview of the diagnostic and therapeutic considerations associated with Amyand’s hernia, ensuring compliance with the latest guidelines and standards in surgical case reporting.

### Case presentation

This is a case of a 30-year-old man with a 2-year history of swelling over his right inguinoscrotal area. The onset of the swelling was insidious, starting slowly and gradually increasing over the 2-year period. The patient noted that the swelling worsened while standing, coughing, and straining, and it reduced when lying down.

The patient’s initial presentation included complaints of agony and irreducible swelling over the right inguinoscrotal area, which persisted for 5 days. There was no significant past medical history of vomiting, nausea, fever, persistent cough, or trauma reported by the patient. Additionally, the patient did not have any known comorbidities, and his regular bowel and urine habits were unaffected.

Upon examination, the patient’s vitals were stable, with no signs of distress. Mild tenderness was noted in the hypogastric and right iliac fossa of the abdominal region. Bowel sounds were within normal limits. Local examination revealed a firm and hard swelling over the right inguinal region, measuring 10×5 cm. The swelling was tender, nonreducible, and demonstrated a positive cough impulse. No signs of discharge or bleeding were observed during the examination.

Further examination revealed an additional soft, non-tender swelling measuring 8×8 cm over the left scrotal region, which was nonreducible. Palpation of bilateral testes was performed separately, with no abnormalities noted.

Ultrasonography was conducted to assess the nature of the swelling. The imaging revealed a complete right inguinoscrotal hernia and bilateral hydrocele with internal echoes. This diagnostic evaluation provided valuable information for planning the subsequent course of action.

The surgery was performed by two senior general surgeons, with assistance from nurses and interns, alongside an anesthesiologist. The anesthesiologist and surgeon worked together to secure the patient’s permission for surgery. A thorough evaluation of the patient’s medical history, clinical examination including the vitals and laboratory findings, such as CBC, BMP, and coagulation tests, was done. The extent of the hernia was disclosed by ultrasound and other imaging methods. A comprehensive methodology was ensured by a cardiac assessment, pulmonary function evaluations, and collaborative decision-making between the surgeon and anesthesiologist.

### Surgical procedure

**Figure 1:**
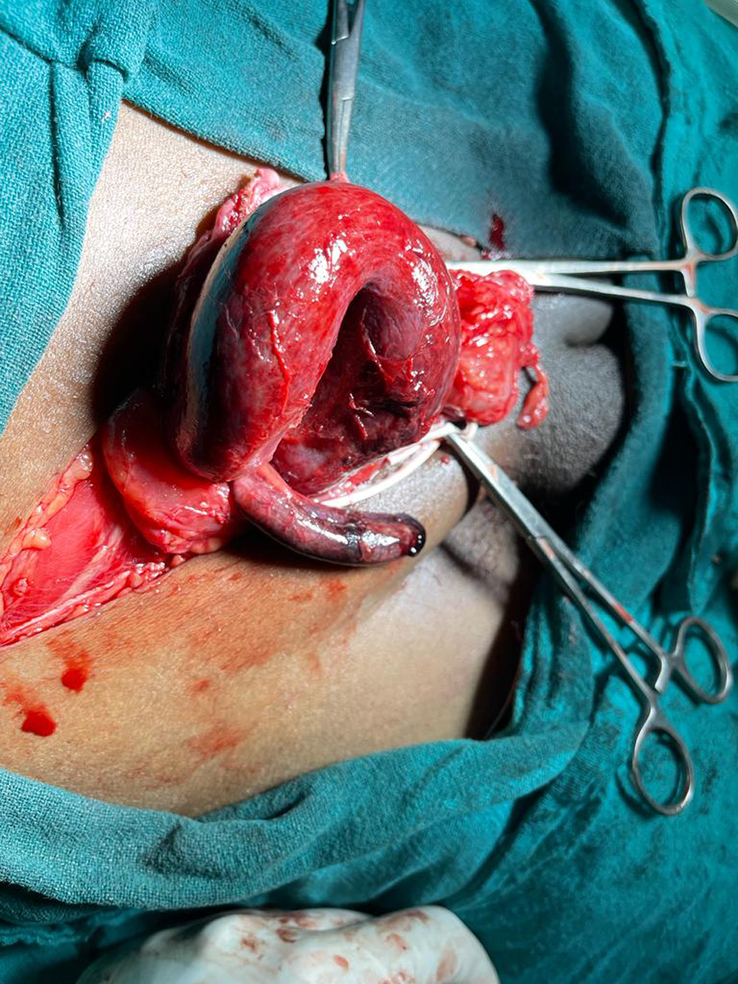
Amyand’s hernia is shown in a figure obtained following surgery, showing a right indirect inguinal hernia with parts of the colon and vermiform appendix inside of it.

During the surgical procedure conducted under general anesthesia, the patient underwent a comprehensive approach addressing a right inguinoscrotal exploration, an appendectomy, and the repair of a right inguinal hernia, employing mesh reinforcement. Upon exploration, it was revealed that a right indirect inguinal hernia contained both the appendix and bowel, necessitating careful dissection of the hernia sac from the surrounding structures and its careful opening to facilitate the subsequent steps. Following the reduction of bowel loops, the appendectomy was performed successfully. Subsequently, a hernial mesh repair was conducted utilizing Vinyl 1-0 to secure the sac and excise any redundant tissue. Prolene 2-0 was employed for closing the defect, while Prolene 1-0 facilitated darning repair. Vicryl 1-0 was then applied to seal the sheath, ensuring structural integrity. Finally, the surgical site was dressed with a sterile covering after the skin was closed using staples. Figure 1 demonstrates an intraoperative view, showcasing the procedure involving the right inguinoscrotal exploration, appendectomy, and hernioplasty, thereby highlighting the presence of both the appendix and bowel within the right indirect inguinal hernia, a scenario often encountered in Type 1 Amyand’s hernia cases.

### Postoperative course

Following the procedure, the patient’s abdomen was neither sore nor swollen. There were no indications of soakage or hematoma at the operative site. The patient’s condition was stable at discharge. The patient underwent regular follow-up appointments at the surgical outpatient department and remained free of symptoms 6 months after the operation.

## Discussion

Amyand’s hernia is an uncommon type of inguinal hernia characterized by the protrusion of the appendix through the inguinal canal and its entrapment within the hernia sac. It predominantly occurs in males and is more commonly observed on the right side of the body^10^. This condition can manifest across a wide age range, from infancy to late adulthood. Younger individuals have a higher likelihood of diagnosis due to the persistence of the processus vaginalis, which predisposes them to herniation^10,11^.

The clinical presentation of Amyand’s hernia can vary widely depending on whether the appendix is inflamed or not. In cases where the appendix is not inflamed, patients may initially present with nonspecific symptoms such as inguinal swelling or discomfort. However, when the appendix becomes inflamed within the hernia sac, it can lead to acute appendicitis, characterized by sudden-onset abdominal pain, fever, nausea, vomiting, and elevated white blood cell count. These symptoms can mimic those of a typical acute appendicitis, making diagnosis challenging. Additionally, patients with Amyand’s hernia may experience complications such as incarceration, strangulation, necrosis, perforation, or rupture of the appendix, which can exacerbate symptoms and lead to severe abdominal pain, tenderness, and signs of peritonitis^12^. In some cases, particularly in infants and young children, the diagnosis may be missed or delayed due to atypical presentation or lack of specific symptoms. Therefore, a high index of suspicion is essential for prompt recognition and appropriate management of Amyand’s hernia^4,13^.

Diagnosing Amyand’s hernia primarily relies on clinical evaluation, although imaging studies like ultrasound and CT scans play a supportive role in confirming the diagnosis and evaluating the extent of appendiceal involvement. A CT scan holds significant importance as it can provide high-resolution images of the hernia sac and the appendix. Moreover, it can effectively differentiate between an inflamed and noninflamed appendix, aiding in the accurate diagnosis of Amyand’s hernia and guiding appropriate treatment decisions^14^. Nevertheless, due to the similarity in symptoms with other hernias and medical conditions, reaching a preoperative diagnosis can pose challenges, often requiring a clinician’s heightened suspicion^15^. Therefore, a comprehensive understanding of the differential diagnosis of inguinoscrotal swelling is crucial. This includes considering other types of hernias, such as direct and indirect inguinal hernias, as well as conditions like testicular torsion and hydroceles. By carefully assessing and distinguishing between these various possibilities, healthcare providers can avoid misdiagnosis and ensure appropriate management strategies are implemented for patients presenting with suspected Amyand’s hernia.

The timing of diagnosing Amyand’s hernia, whether preoperatively or during surgery, has significant implications for patient management. Preoperative identification allows for better surgical planning, minimizing intraoperative surprises and facilitating precise decision-making regarding the extent of intervention needed. Conversely, diagnosing Amyand’s hernia intraoperatively can complicate procedures, potentially requiring unplanned modifications and prolonging surgical time. Unexpected findings may necessitate additional interventions, increasing the risk of complications and challenging the surgeon’s preparedness.

The management of Amyand’s hernia is guided by various factors including clinical presentation, severity of inflammation, and patient-specific characteristics. The Losanoff and Basson classification system provides a structured framework for surgeons to make informed treatment decisions based on the level of appendiceal inflammation. Lossannof and Basson introduced four classifications of Amyand’s hernias that offer valuable insights into treatment strategies tailored to the appendix pathology^13^. Type 1 denotes a normal appendix and typically involves tension-free hernia repair without appendectomy, as the operation is considered clean and carries a low risk of infection^16^. Type 2 Amyand’s hernias present with acute nonperforated appendicitis, where appendectomy alongside hernia repair is recommended. Type 3 involves acute appendicitis with perforation and intra-abdominal sepsis, necessitating more extensive surgical intervention including laparotomy, appendectomy, and primary hernia repair with irrigation. Finally, type 4 is characterized by acute appendicitis complicated by other intra-abdominal pathologies, such as incidental masses or tumors.

In our case, the patient presented with a 2-year history of right inguinoscrotal swelling, ultimately diagnosed intraoperatively as an Amyand’s hernia, type 1. Given the patient’s clinical profile and the nature of the hernia, which involved a normal appendix, we opted for appendectomy during the surgery. This decision was based on several factors, including the patient’s age, overall health status, and potential tolerance to retaining a noninflamed appendix. Additionally, considering the surgical setting and the need to prevent future complications, removal of the appendix was deemed appropriate. The choice of appendectomy in our case was further supported by recommendations from Rikki *et al*.^3^, advocating for appendicectomy in all cases of Amyand’s hernia, ensuring comprehensive management of the condition.

Beyond the surgical intervention, ongoing management of Amyand’s hernia involves addressing concerns regarding unintentional appendectomy and adapting to evolving surgical techniques. There is also a need to recognize the possibility of tissue weakness and recurrence following appendectomy surgery, highlighting the importance of a tailored care plan^17^. Factors such as the degree of inflammation, presence of abdominal sepsis, and any underlying comorbidities must be carefully considered in managing these cases^18^. Moreover, recent research indicating a link between Amyand’s hernia and appendiceal cancer underscores the significance of postoperative vigilance and thorough evaluation in every case^19^. Each instance requires meticulous consideration to ensure optimal outcomes and minimize potential complications.

In conclusion, Amyand’s hernia presents unique diagnostic and management challenges due to its rarity and variable clinical presentation. A multidisciplinary approach involving clinical evaluation, diagnostic imaging, and surgical expertise is crucial for achieving optimal outcomes in patients with this uncommon condition. Further research in the field of Amyand’s hernia is essential to address existing gaps and refine management strategies. While traditional guidelines advise against mesh usage in cases involving an inflamed or perforated appendix, recent studies suggest the potential efficacy of mesh repair even in acute appendicitis cases without an increased risk of infection provided that the operative field remains uncontaminated^6,20^. Despite advancements, controversies persist regarding the management of noninflamed appendix found in Amyand’s hernia and the necessity of mesh utilization postappendectomy in Type 2 Amyand’s hernia scenarios. Addressing these issues through robust research endeavors will contribute to a better understanding of Amyand’s hernia and optimize patient outcomes.

## Conclusion

In summary, the case of Amyand’s hernia presented herein exemplifies the diagnostic and therapeutic considerations inherent to this rare condition. Through meticulous preoperative assessment and surgical intervention, successful management was achieved, culminating in the resolution of the patient’s symptoms and restoration of health. This case underscores the importance of maintaining a high index of suspicion, particularly in the context of inguinoscrotal pathology, to facilitate timely diagnosis and intervention. Furthermore, it emphasizes the critical role of comprehensive surgical planning and precise execution in optimizing patient outcomes. Importantly, this case report contributes significantly to the existing literature by providing detailed insights into the diagnostic challenges and surgical management strategies associated with Amyand’s hernia. By disseminating this information, healthcare practitioners can enhance their awareness and expertise in managing similar cases, ultimately improving patient care and prognosis. Moving forward, continued vigilance and collaborative efforts among healthcare professionals are essential to ensure early recognition and effective management of Amyand’s hernia, thereby further advancing the field of surgical practice.

## Methods

This case report has been reported in line with the SCARE 2023 criteria as outlined in the SCARE 2023 guideline^21^.

## Ethical approval

The collection and evaluation of all patient health information was performed in a Health Insurance Portability and Accountability Act (HIPAA) – compliant manner.

## Patient consent

Written informed consent was obtained from the patient for publication and any accompanying images. A copy of the written consent is available for review by the Editor-in-Chief of this journal on request.

## Sources of funding

The author(s) received no financial support for the research, authorship and/or publication of this case report.

## Author contribution

All authors have equally contributed to this case report.

## Conflicts of interest disclosure

The authors declare no conflicts of interest relevant to this article. No financial or nonfinancial interests have influenced the development of this work.

## Research registration unique identifying number (UIN)

Not applicable.

## Guarantor

No Guarantor was involved.

## Data availability statement

Not applicable.

## Provenance and peer review

Not commissioned.
